# Ancoats Hospital and the War

**Published:** 1915-02-20

**Authors:** 


### Ancoats Hospital and the War.

WHEN war was declared Ancoats Hospital was faced with very grave difficulties. Out of the honorary medical and surgical staff three were called up for active service. Captain W. R. Douglas, F.R.C.S., senior honorary surgeon, left for Egypt with the Lancashire Territorials, and is now stationed at Alexandria as surgeon to the military hospital there. For some time after his arrival he was attached to the Suez Canal Defence Force stationed at Ismailia, and has recently been appointed examiner in surgery to the Egyptian Government for the State examination in medicine. Lieutenant John Morley, F.R.C.S., honorary surgeon to Ancoats Hospital, also left for Egypt with the Lancashire Territorials, and is now surgeon to the military hospital at Khartoum. Lieutenant John Ward, assistant physician to the hospital, left immediately on the outbreak of hostilities, and is looking after the "Boys" stationed at Heliopolis, near Cairo. From the resident staff, Drs. Marshall and Whitehead received commissions in the Territorials, and are now doing their duty to the country by keeping the soldiers training at Southport in an efficient condition. Lieutenant Bedale, late house surgeon, is now in full charge of the military hospital at Cyprus, and is no doubt profiting by the experience gained while at Ancoats Hospital in the treatment of the 20,000 accident cases which pass through this hospital per annum. Dr. F. B. Smith, who was appointed house surgeon on the departure of Dr. Bedale, has recently received his commission, and is now also with the Territorials at present training at Southport. These doctors, having answered their country's call, left Ancoats Hospital in a very difficult position, especially as the civil population continued to apply for treatment in as large numbers as ever. The present honorary staff consists of Captain D. E. Core, physician; Dr. C. E. Lea, physician; Mr. Harry Platt, F.R.C.S., M.S., surgeon; Mr. Gilbert Warburton, F.R.C.S., surgeon; Captain W. J. S. Bythell, in charge of the *X*-ray Department; while the resident staff include Mr. J. R. Slack, house surgeon; Dr. Booth, house physician; Mr. Brook; and Mr. Christopher. At the commencement of the war also six sisters, who were Territorial nurses, were called up to attend to the soldiers at the military hospitals to which their unit was attached, and since then three more nurses and sisters have left to nurse the wounded. In November last the committee placed at the disposal of the military authorities twenty beds at the hospital at Mill Street, and thirty-one beds at the Convalescent Home at Alderley Edge, and up to the end of January 127 wounded soldiers have been admitted.

